# Impact of a case management model on maternal and neonatal outcomes in gestational hypertension: a randomized controlled trial

**DOI:** 10.3389/fgwh.2026.1677541

**Published:** 2026-03-20

**Authors:** Yaping Tang, Muli Shi, Chunfeng Liu, Hongli Huang, Jinlian Bao

**Affiliations:** Shenzhen Luohu District Maternal and Child Health Hospital, Shenzhen, Guangdong, China

**Keywords:** blood pressure control, case management model, gestational hypertension, neonatal outcomes, pregnancy complications

## Abstract

**Objective:**

This study aimed to evaluate the effectiveness of a case management model in the comprehensive management of gestational hypertension, focusing on its impact on blood pressure control, pregnancy complications, neonatal health, and maternal satisfaction.

**Methods:**

A randomized controlled trial was conducted, enrolling 100 pregnant women with hypertension who attended the high-risk outpatient clinic of our hospital from March 2023 to March 2025. Participants were randomly assigned to either an experimental group (*n* = 50), which received a case management model intervention, or a control group (*n* = 50), which received standard prenatal care. The two groups were compared regarding blood pressure control, incidence of pregnancy complications, gestational age at delivery, preterm birth rate, birth weight, low birth weight incidence, neonatal transfer rate, and maternal satisfaction. Statistical analyses were conducted using SPSS 23.0, with a significance level of *P* < 0.05.

**Results:**

The experimental group showed significantly better blood pressure control, with lower systolic and diastolic blood pressure levels compared to the control group (*P* < 0.05). The incidence of pregnancy complications, such as preeclampsia, placental abruption, and Hemolysis, Elevated Liver Enzymes, and Low Platelet count (HELLP) syndrome, was significantly reduced in the experimental group (*P* < 0.05). Additionally, the experimental group had an extended gestational age at delivery, a lower preterm birth rate, and higher birth weight (*P* < 0.05). Neonatal outcomes were improved, as evidenced by a significant reduction in the incidence of low birth weight and neonatal transfer rates (*P* < 0.05). Maternal satisfaction with care was also significantly higher in the experimental group (*P* < 0.01).

**Conclusion:**

The case management model significantly improved maternal and neonatal outcomes by enhancing blood pressure control, reducing pregnancy complications, and increasing maternal satisfaction. These results support the integration of case management into standard care for pregnant women with hypertension to optimize pregnancy outcomes.

## Introduction

Gestational hypertension is a significant global health issue, affecting an estimated 5%–8% of all pregnancies (1). In China, the incidence of gestational hypertension is reported at approximately 7.3%, with regional variations ranging from 1.6% to 11.3% (2). These disorders encompass a range of conditions, including gestational hypertension, preeclampsia, and eclampsia, all of which contribute to elevated maternal and fetal morbidity and mortality (3). Globally, gestational hypertension is one of the leading causes of maternal mortality and is a major contributor to preterm births, low birth weight, and neonatal intensive care unit (NICU) admissions (4). The consequences of gestational hypertension are particularly concerning when considering perinatal mortality rates. In China, perinatal mortality rates associated with gestational hypertension are approximately 3.59%, with stillbirths accounting for over 80% of these outcomes (5). These statistics highlight the urgent need for more effective management strategies to reduce the maternal and neonatal burden associated with gestational hypertension. Despite advancements in prenatal care, gestational hypertension remains a leading cause of adverse pregnancy outcomes, which emphasizes the importance of developing innovative and comprehensive management models (6). Current management protocols for gestational hypertension focus on regular monitoring and pharmacological interventions, including antihypertensive medications such as methyldopa, nifedipine, and labetalol, as well as magnesium sulfate for seizure prevention in cases of preeclampsia (7). However, these approaches often fall short of addressing the full spectrum of care needed, as they do not consistently integrate lifestyle modifications, such as dietary changes and physical activity, which are essential for optimal hypertension control (8). Furthermore, the lack of personalized care and ongoing support can lead to gaps in treatment adherence and poor health outcomes for both mothers and infants. Lifestyle interventions, including low-sodium diets, regular exercise, and consistent blood pressure monitoring, have been shown to improve hypertension control during pregnancy (9). However, these modifications require ongoing support and education, which are not always adequately provided in conventional care settings. Therefore, there is a growing need for more individualized and holistic approaches to managing gestational hypertension, which incorporate both medical treatment and personalized lifestyle interventions. This study aims to assess the effectiveness of a case management model designed to provide comprehensive care for pregnant women with hypertension. The case management model focuses on individualized treatment plans that include regular follow-ups, health education, dietary and exercise guidance, and daily blood pressure monitoring. By evaluating the impact of this model on key outcomes such as blood pressure control, complication rates, neonatal health, and maternal satisfaction, we aim to determine whether this approach can improve the overall management of gestational hypertension and reduce associated risks. We hope to provide valuable insights into how a more integrated and supportive management model can lead to better maternal and neonatal outcomes. By emphasizing the importance of personalized care and ongoing support, this study seeks to contribute to the development of more effective strategies for managing gestational hypertension in clinical settings.

## Materials and methods

This randomized controlled trial was not prospectively registered in a public clinical trial registry. At the time of study initiation, prospective registration was not mandated by the local ethics committee for single-center, non-pharmacological care-model intervention studies. The study was ethically approved by the Shenzhen Luohu District Maternal and Child Health Hospital Ethics Committee (Approval Number: LI2023110750). Written informed consent was obtained from all participants prior to participation, acknowledging that their data might be used for research and publication purposes.

### Study design and participants

A total of 100 pregnant women with hypertension who visited the high-risk management clinic of our hospital between March 1, 2023, and March 31, 2025, were enrolled in the study. Participants were randomly assigned into two groups: the experimental group (ExGp) and the control group (CnGp), with 50 participants in each.

Random allocation was performed using a computer-generated random number sequence prepared by an independent research assistant not involved in participant recruitment or intervention delivery. Allocation concealment was ensured using sealed, opaque, sequentially numbered envelopes, which were opened only after enrollment. Due to the nature of the case management intervention, blinding of participants and care providers was not feasible. However, outcome assessment and statistical analysis were conducted by investigators who were blinded to group allocation. The control group received standard high-risk pregnancy management, while the experimental group received an additional case management model to evaluate its effectiveness in improving maternal and neonatal outcomes. An *a priori* sample size calculation was performed based on systolic blood pressure as the primary outcome. Assuming a mean between-group difference of 6–8 mmHg with a standard deviation of 10–12 mmHg, a two-sided independent samples *t*-test with an *α* level of 0.05% and 80% power indicated that at least 45 participants were required per group. To account for potential attrition, the target sample size was increased to 50 participants per group, resulting in a total sample size of 100 pregnant women.

### Inclusion and exclusion criteria

Pregnant women were included in the study if they exhibited abnormal blood pressure readings (higher-systolic) in the same arm on at least two consecutive prenatal check-ups and had one or more high-risk factors for hypertension (10). Additionally, the presence of abnormal 24-h urine protein levels, a diagnosis of chronic hypertension before or during pregnancy, and adherence to regular prenatal check-ups and follow-ups. Furthermore, participants were required to have a high school education or higher and be capable of communicating effectively via WeChat (Chinese social platform). Informed consent was obtained from all enrolled individuals. While, the patient Excluded who refusal to sign the informed consent form, poor compliance due to conditions such as malignancy, mental illness, or communication disorders, and engagement in harmful behaviors such as alcohol consumption, smoking, or drug abuse. Participants who withdrew from the study before its completion were also excluded from the final analysis.

### Intervention and study procedures

Participants in the control group received standard high-risk pregnancy management, which included routine prenatal examinations, health education, calcium (calcium carbonate 600 mg/day) and multivitamin supplementation (including folic acid 400–800 µg/day), and low-dose aspirin therapy (75–150 mg/day). Pharmacological interventions such as methyldopa, nifedipine, or labetalol for hypertension; magnesium sulfate for seizure prophylaxis in preeclampsia; and diazepam or phenytoin for seizure management were administered as clinically indicated (11). In contrast, participants in the experimental group received an individualized case management model in addition to conventional care. A designated case manager developed personalized treatment plans based on each participant's clinical condition. Development of personalized management plans: Individualized case management plans were formulated at enrollment based on a comprehensive assessment that included gestational age, baseline systolic and diastolic blood pressure, body mass index, presence of comorbidities (gestational diabetes), prior obstetric history, and laboratory indicators such as urine protein levels. Based on this assessment, the case manager, in consultation with an obstetrician, defined individualized targets for blood pressure control, dietary modification, physical activity, and follow-up intensity. Tools and monitoring: Participants were provided with or instructed to use validated automatic home blood pressure monitors. Blood pressure measurements were recorded twice daily (morning and evening) and uploaded through a dedicated WeChat-based platform. Educational materials, including videos, infographics, and written guidance on hypertension management during pregnancy, were disseminated through the platform. Follow-up frequency and mode: Follow-up was conducted through a combination of remote and in-person approaches. Daily blood pressure data were reviewed by the case management team, with weekly scheduled online consultations via WeChat or telephone. In-person outpatient visits were arranged according to routine prenatal schedules or earlier if abnormal blood pressure readings or clinical symptoms were identified. Medication adjustments, lifestyle counseling, and psychological support were provided as needed throughout the intervention period. This model included dietary recommendations, such as a balanced, low-sodium diet (no more than 2,300 mg of sodium per day) rich in fruits, vegetables, and whole grains, with an emphasis on increasing dietary intake of potassium (e.g., bananas, oranges) and calcium (e.g., dairy products, fortified plant-based milks), while limiting caffeine and sugar intake. Exercise recommendations involved moderate-intensity physical activity, such as daily walking (30 min per day) or low-impact aerobic exercises (stationary cycling) for at least 5 days per week, tailored to the participant's condition and fatigue levels. Participants were also instructed to measure their blood pressure twice daily (morning and evening) using a validated automatic blood pressure cuff, with target blood pressure goals of maintaining systolic pressure below 140 mmHg and diastolic pressure below 90 mmHg. The case manager provided ongoing adherence support by ensuring compliance with prescribed antihypertensive medications (such as labetalol or nifedipine), calcium and multivitamin supplements, and lifestyle modifications, with weekly check-ins via phone or online consultations to discuss adherence, address concerns, and offer encouragement.

### WeChat-based nutrition education and monitoring

Upon entering the intervention stage, participants in the experimental group were enrolled in a dedicated WeChat group, which was established following the formation of a structured intervention plan. Each participant received an individualized evaluation sheet, and daily blood pressure readings were uploaded to the group. Obstetricians and gynecologists actively participated in the WeChat group, overseeing implementation, monitoring adherence, and providing necessary guidance and support. Prior to initiating the intervention, trust was established with the pregnant women and their families to ensure engagement and cooperation. A core intervention team was formed, comprising three key members: senior obstetricians and gynecologists, case managers, and obstetric nurses. All researchers were briefed on the study objectives, methodology, and specific intervention components during a preparatory meeting, and all required materials were organized in advance. The intervention was conducted from the hypertension detection to until delivery, with postpartum follow-up managed by the Women's Health Department. The intervention strategy included:

Only Based Monitoring and Education: High-risk participants were invited to join the WeChat group, which was managed by the research team. Educational materials, including instructional videos and infographics on perinatal health, were disseminated 1–2 times per week. If consultation results were unavailable on Mondays and Wednesdays, participants could upload their inquiries to the WeChat group for online medication guidance, reducing unnecessary hospital visits.

Offline Hypertension Education: Regular in-person educational sessions were held to improve knowledge and self-management of hypertension during pregnancy.

Daily Blood Pressure Monitoring and Medical Guidance: Participants uploaded their blood pressure readings daily to the WeChat group. Medical staff provided timely feedback, medication adjustments, and dietary recommendations to optimize blood pressure control.

### Social and family support

For participants with inadequate blood pressure control, direct communication was maintained to provide personalized support. Emotional and psychological needs were addressed by actively listening to participants concerns. Family members were encouraged to offer companionship and motivation, fostering a supportive environment to enhance self-management, boost confidence in childbirth, and promote adherence to the intervention plan.

### Outcome measures

The study evaluated both maternal and neonatal outcomes to assess the effectiveness of the intervention. Maternal outcomes included blood pressure control, measured as the mean systolic and diastolic blood pressure throughout pregnancy, and the incidence of pregnancy complications such as preeclampsia and eclampsia. Gestational age at delivery was recorded to determine whether the intervention influenced pregnancy duration. Neonatal outcomes included birth weight, with a specific focus on the prevalence of low birth weight, and the rate of preterm birth. The neonatal transfer rate, defined as the proportion of newborns requiring admission to the neonatal intensive care unit (NICU), was also analyzed. Additionally, maternal satisfaction with the intervention was assessed using a structured questionnaire, evaluating perceptions of care quality, accessibility of medical guidance, and overall confidence in managing pregnancy-related hypertension.

### Statistical analysis

All statistical analyses were performed using SPSS 23.0 software. Continuous variables were expressed as mean ± standard deviation (SD) and analyzed using the independent *t*-test. Categorical variables were presented as frequencies and percentages and compared using the *χ*^2^ test. The normality of continuous variables was assessed using the Shapiro–Wilk test. Variables with normal distribution were analyzed using independent samples *t*-tests. For categorical variables, the *χ*^2^ test was applied when all expected cell counts were ≥5; Fisher's exact test was used when expected cell counts were <5. A *p*-value < 0.05 was considered statistically significant.

## Results

### General information of the participants

A total of 100 pregnant women with hypertension participated in this study, with 50 women in the experimental group (ExGp) and 50 in the control group (CnGp). The baseline characteristics of both groups were comparable in terms of age, gestational age at enrollment, and hypertension history, with no statistically significant differences observed (*P* > 0.05), as shown in Table 1. Regarding high-risk factors, the distribution was as follows: 44% of participants in the ExGp were aged 35 years or older, compared to 56% in the CnGp. Obesity was observed in 30% of the ExGp participants and 36% of the CnGp participants. Gestational diabetes mellitus (GDM) occurred in 18% of the ExGp participants and 24% in the CnGp. Other high-risk factors were present in 16% of the ExGp and 20% of the CnGp participants. These differences in high-risk factors were not statistically significant (*P* > 0.05).

**Table 1:** Demographic and Clinical Characteristics of Participants in the Experimental and Control Groups.

Items	Experimental group (*n* = 50)	Control group (*n* *=* 50)	Statistics	*P* value
Age (Years)	31.2 ± 4.9	32.5 ± 5.8	*t* = 1.21	0.231
BMI (kg/m^2^)	24.1 ± 3.7	25.6 ± 4.3	*t* = 1.85	0.068
Pregnancy Times	2.1 ± 1.1	2.3 ± 1.2	*t* = 0.89	0.378
High Risk Factors			*χ*^2^ = 4.56	1.102
High Age (≥35 years)	22 (44.0)	28 (56.0)		
Obesity	15 (30.0)	18 (36.0)		
GDM	9 (18.0)	12 (24.0)		
Other	8 (16.0)	10 (20.0)		

GDM, gestational diabetes mellitus; BMI, body mass index.

### Incidence of pregnancy complications and preterm birth

The incidence of preterm birth in the experimental group was significantly lower than in the control group, with a reduction of 15% (*P* < 0.05). Specifically, 8 participants (16%) in the experimental group experienced preterm birth, compared to 15 participants (30%) in the control group. The difference between the groups was statistically significant (*χ*^2^ = 4.55, *P* = 0.033) (Table 2**)**. When pregnancy complications were analyzed collectively, the overall incidence was significantly different between the two groups (*χ*^2^ = 5.78, *P* = 0.016). The distribution of individual complications, including preeclampsia, severe preeclampsia, placental abruption, and HELLP syndrome, is presented in Table 2. These findings indicate that the case management model was associated with improved pregnancy outcomes, particularly through a significant reduction in preterm birth.

**Table 2:** Comparison of the incidence of preterm birth and pregnancy complications between the experimental and control groups.

Items	Experimental group (*n* *=* 50)	Control group (*n* *=* 50)	*χ*^2^ Value	*P* Value
Preterm Birth (<37 weeks)	8 (16.0)	15 (30.0)	4.55	0.033
Pregnancy complications			5.78	0.016
Preeclampsia	22 (44.0)	14 (28.0)		
Severe preeclampsia	10 (20.0)	5 (10.0)		
Placental abruption	6 (12.0)	2 (4.0)		
HELLP Syndrome	3 (6.0)	1 (2.0)		

HELLP, hemolysis; elevated liver enzymes, and low platelet count.

### Blood pressure control and termination of pregnancy

Blood pressure control was significantly improved in the experimental group compared to the control group. The systolic blood pressure in the experimental group was significantly lower (127.8 ± 10.6 mmHg) than that in the control group (135.2 ± 12.4 mmHg), with a statistically significant difference (*t* = 3.25, *P* = 0.002). Similarly, the diastolic blood pressure in the experimental group (82.3 ± 7.5 mmHg) was significantly lower than in the control group (88.7 ± 8.9 mmHg) (*t* = 3.98, *P* = 0.001), indicating better control of hypertension in the experimental group (Table 3**)**. The gestational age at delivery was significantly longer in the experimental group, with a mean gestational age at termination of 38.4 ± 1.8 weeks, compared to 37.6 ± 2.1 weeks in the control group. This difference was statistically significant (*t* = 2.11, *P* = 0.038). Additionally, the incidence of premature birth was lower in the experimental group, confirming that the case management model contributed to better management of hypertensive disorders during pregnancy and improved pregnancy outcomes. To further quantify the magnitude of these effects, effect size estimates were calculated. The between-group mean difference in systolic blood pressure was −7.4 mmHg, corresponding to a moderate effect size (Cohen's *d* = 0.63), while the mean difference in diastolic blood pressure was −6.4 mmHg (Cohen's *d* = 0.77). These reductions are clinically meaningful, as even modest decreases in blood pressure during pregnancy are associated with reduced risks of hypertensive complications and adverse perinatal outcomes. In addition, the prolongation of gestational age by approximately 0.8 weeks in the experimental group represents a clinically relevant improvement, particularly in reducing the risk of preterm birth.

**Table 3:** Comparison of blood pressure during pregnancy and termination of pregnancy between the experimental and control groups.

Items	Experimental group (*n* *=* 50)	Control group (*n* *=* 50)	*t* value	*P* value
Systolic blood pressure (mmHg)	127.8 ± 10.6	135.2 ± 12.4	3.25	0.002
Diastolic blood Pressure (mmHg)	82.3 ± 7.5	88.7 ± 8.9	3.98	0.001
Termination of pregnancy (weeks)	38.4 ± 1.8	37.6 ± 2.1	2.11	0.038

### Neonatal outcomes

The neonatal outcomes of the experimental group showed significant improvements. The birth weight of neonates was significantly higher in the experimental group compared to the control group, with an average increase of 200 g (*P* < 0.05). Furthermore, the incidence of low-birth-weight infants was significantly lower in the experimental group (*P* < 0.05). The rate of neonatal transfer to the neonatal intensive care unit (NICU) was also significantly reduced in the experimental group, with a decrease of 12% (*P* < 0.05), as shown in Table 4.

**Table 4:** Comparison of fetal birth weight and neonatal outcomes between the experimental and control groups.

Items	Experimental group (*n* *=* 50)	Control group (*n* *=* 50)	Statistics	*P* value
Fetal birth weight (g)	3,250 ± 380	3,050 ± 450	*t* = 2.43	0.017
Low birth weight (<2,500 g)	6 (12.0)	12（24.0）	*t* = 3.24	0.042
Neonatal Outcome			χ^2^ ^=^ 4.12	0.042
Transfer to neonatology department	10 (20.0)	18 (36.0)		
Accompany with Mother	40 (80.0)	32 (64.0)		

### Patient satisfaction

Patients in the experimental group reported significantly higher satisfaction with the case management model compared to the control group. The overall satisfaction rate was 86% in the experimental group, while only 62% of patients in the control group expressed satisfaction (*P* = 0.007) (Table 5). Of 70% of patients in the experimental group were very satisfied, compared to 42% in the control group. Additionally, 16% of the experimental group and 20% of the control group were satisfied. Only 4% of patients in the experimental group were dissatisfied, compared to 12% in the control group. Patients in the experimental group also reported feeling more confident in managing their hypertension and experiencing better access to care, contributing to their higher levels of satisfaction. The case management model implemented in this study showed significant improvements in both maternal and neonatal outcomes. The experimental group demonstrated better blood pressure control, a longer gestational age, and a lower incidence of pregnancy complications and preterm birth. Neonatal outcomes were also improved, with higher birth weights and fewer neonatal transfers to the NICU. These results highlight the positive impact of the case management model on both patient satisfaction and overall care outcomes for pregnant women with hypertension.

**Table 5:** Comparison of maternal satisfaction between the experimental and control groups.

Groups	Very satisfied	Satisfied	Average	Dissatisfied	Overall satisfaction rate
Experimental Group (*n* = 50)	35 (70.0)	8 (16.0)	5 (10.0)	2 (4.0)	43 (86.0)
Control Group (*n* = 50)	21 (42.0)	10 (20.0)	13 (26.0)	6 (12.0)	31 (62.0)
χ^2^ value					7.23
*P* value					0.007

## Discussion

Gestational hypertension is a prevalent complication of pregnancy that poses significant risks to both maternal and neonatal health and its global challenge. The increasing global incidence of gestational hypertension presents a serious public health challenge (12).

Unlike routine prenatal care, which often focuses on episodic clinical monitoring, the present study evaluated a structured case management model that integrates continuous supervision, lifestyle modification, and digital health support. We demonstrated that women receiving case management achieved better blood pressure control, significantly lower preterm birth rates, improved neonatal outcomes, and higher satisfaction (*P* < 0.05), compared with standard care. These results validate the effectiveness of the case management model and offer a strong foundation for optimizing management strategies for gestational hypertension (13).

The core strengths of the case management model lie in its personalized, comprehensive, and multidisciplinary approach. Case managers tailored prenatal care plans based on individual risk factors such as blood pressure levels, BMI, and other high-risk conditions. This approach enabled continuous health education and psychological support through WeChat groups, offline patient education, and other interactive methods. Similar to the work of Ernawati et al. and Dailah et al., our study highlights that individualized education, regular monitoring, and timely feedback are key mechanisms driving improved self-management and clinical outcomes (14, 15). Pregnant women in the experimental group uploaded daily blood pressure data, and case managers, in consultation with obstetricians, adjusted medication regimens and lifestyle recommendations in real time. This dynamic management approach effectively minimized fluctuations in blood pressure. Additionally, the case management model integrated resources from multiple disciplines, including nutritionists, psychologists, and internal medicine specialists. This patient-centered, multidisciplinary collaboration reduced the incidence of complications such as preeclampsia and placental abruption (*P* < 0.05). By fostering stronger social support systems, such as family involvement and peer education (16), the case management model further improved patient compliance. This is consistent with research by Peng Yumei et al. on the importance of continuous supervision and emotional support in managing gestational diabetes (17, 18). However, evidence specifically targeting gestational hypertension remains limited, and most prior studies have focused on pharmacological management or isolated lifestyle interventions rather than integrated care models. A notable strength of our study is the combination of online (WeChat-based) and offline interventions, which allowed real-time blood pressure monitoring, rapid clinical feedback, and continuous patient engagement. This hybrid approach extends previous digital health studies by embedding remote monitoring within a coordinated case management framework, rather than using digital tools in isolation. During pregnancy a period characterized by rapid physiological changes and heightened anxiety this continuous support may explain the observed improvements in blood pressure stability and patient confidence. Comparable digital-supported interventions have reported mixed results, often limited by low adherence or lack of clinical integration, underscoring the importance of the multidisciplinary coordination applied in our model. In contrast to some observational studies suggesting reductions in specific hypertensive complications, our results indicate that the primary benefit of case management lies in overall pregnancy outcome optimization, particularly through reduced preterm birth, rather than uniform reductions across all individual complication subtypes. Importantly, the observed benefits of the case management model were not only statistically significant but also clinically meaningful. The reductions in systolic and diastolic blood pressure observed in the experimental group are of a magnitude known to reduce the risk of progression to severe hypertensive disorders, preterm birth, and adverse neonatal outcomes. Likewise, the prolongation of gestational age and reduction in preterm birth represent clinically relevant improvements with direct implications for neonatal survival and long-term health. These findings emphasize that the intervention achieved meaningful improvements beyond statistical significance alone. This distinction is important and highlights the complexity of hypertensive disorders during pregnancy, which are influenced by multiple biological and contextual factors. Future studies should explore whether earlier initiation or longer duration of case management could further modify complication profiles.

By optimizing resource allocation and reducing unnecessary hospital visits, this model has the potential to improve efficiency while maintaining patient-centered care, aligning with national strategies such as the “Healthy China 2030” initiative. Additionally, this model could reduce healthcare costs by optimizing resource allocation, including minimizing unnecessary hospitalizations. Nevertheless, implementation challenges remain, including the shortage of formally trained case managers and the absence of standardized certification systems, as well as concerns related to data privacy when using social media–based platforms. Development of secure, hospital-integrated digital systems and standardized training programs for nurse case managers will be essential for wider adoption, as seen in the Spain model (19). Another challenge is the risk of data privacy breaches, particularly with platforms like WeChat. Future development of an intelligent, secure management platform that integrates electronic medical records and remote monitoring is crucial. Additionally, regional disparities in healthcare quality must be considered. This study was conducted at a specialized maternal and child health hospital, and its applicability to general hospitals and primary healthcare settings needs further validation. One possible solution is the integration of high-quality resources through medical alliances and the design of tailored management strategies for institutions at different levels.

Although this study demonstrates the effectiveness of a case management model in improving maternal and neonatal outcomes among women with gestational hypertension, several limitations should be acknowledged. First, the single-center design and relatively modest sample size may limit the generalizability of the findings to other regions, healthcare systems, and populations with differing sociodemographic characteristics or levels of healthcare resources. The intervention was implemented in a specialized maternal and child health hospital with established digital infrastructure and trained case managers, which may not be directly replicable in all clinical settings. Therefore, caution is warranted when extrapolating these results to primary healthcare institutions or resource-limited environments. Future large-scale, multicenter randomized controlled trials with more diverse populations are needed to confirm the robustness and external validity of the present findings. In addition, the follow-up period was limited to 42 days postpartum, precluding evaluation of long-term maternal and child health outcomes, such as future cardiovascular or metabolic risk. Longer follow-up studies will be important to assess the sustained impact of case management across the life course. The intervention integrated digital blood pressure monitoring, structured patient education, lifestyle modification, and psychosocial support delivered through both online and offline modalities. While this comprehensive approach reflects real-world clinical practice and may explain the overall effectiveness observed, it limits the ability to isolate the independent contribution of each individual component. The present study did not include an economic evaluation; nevertheless, the observed improvements in pregnancy outcomes and reductions in preterm birth suggest that the case management model may have potential cost-saving benefits, which warrant formal health economic analysis in future research. With ongoing advances in digital health and artificial intelligence, integration of machine-learning–based risk prediction tools into case management frameworks may further enhance individualized care and early intervention. From a policy perspective, these findings offer practical insights for strengthening maternal and child health services, in line with the objectives of the “14th Five-Year National Health Plan.” Incorporating case management into clinical guidelines for hypertension during pregnancy could support more patient-centered, efficient care delivery. Socially, the case management approach not only improved maternal and neonatal outcomes but also enhanced family health literacy and maternal self-efficacy, consistent with the World Health Organization's health promotion framework. Notably, the high acceptance of the WeChat-based intervention among low-income participants highlights its feasibility and scalability, supporting its potential as a widely applicable health management strategy.

## Conclusion

Our case management model significantly improved the blood pressure control level of patients with hypertension during pregnancy, reduced the risk of maternal and infant complications, and improved patient satisfaction through personalized intervention, multidisciplinary collaboration and full-process management. Although there are challenges in human resources and information construction in the promotion, its clinical value and social benefits cannot be ignored. In the future, it is necessary to promote the application of this model in a wider range of scenarios through policy support, technical empowerment and standardized training, and ultimately achieve quality and efficiency improvement in the management of hypertension during pregnancy.

## Data Availability

The original contributions presented in the study are included in the article/Supplementary Material, further inquiries can be directed to the corresponding author.

## References

[1] Khedagi AM Bello NA. Hypertensive disorders of pregnancy. Cardiol Clin. (2021) 39(1):77–90. 10.1016/j.ccl.2020.09.005 33222817 PMC7720658

[2] Li F Qin J Zhang S Chen L. Prevalence of hypertensive disorders in pregnancy in China: a systematic review and meta-analysis. Pregnancy Hypertens. (2021) 24:13–21. 10.1016/j.preghy.2021.02.001 33626437

[3] Braunthal S Brateanu A. Hypertension in pregnancy: pathophysiology and treatment. SAGE Open Med. (2019) 7:2050312119843700. 10.1177/2050312119843700 31007914 PMC6458675

[4] Wang L Cheng L Zhang S Su M Jin Y Luo D. Mediation effect of pregnancy-induced hypertension on the association between assisted reproductive technology and adverse neonatal outcomes: a population-based study. BMC Pregnancy Childbirth. (2023) 23:385. 10.1186/s12884-023-05694-3 37231502 PMC10210490

[5] Lyu X Zhang W Zhang J Wei Y Guo X Cui S Morbidity and maternal and infant outcomes of hypertensive disorder in pregnancy in China in 2018. J Clin Hypertens (Greenwich). (2021) 23(6):1194–204. 10.1111/jch.14248 33788388 PMC8678747

[6] Garovic VD Dechend R Easterling T Karumanchi SA McMurtry Baird S Magee LA Hypertension in pregnancy: diagnosis, blood pressure goals, and pharmacotherapy. Hypertension. (2022) 79(2):e21–41. 10.1161/HYP.0000000000000208 34905954 PMC9031058

[7] Brown CM Garovic VD. Drug treatment of hypertension in pregnancy. Drugs. (2014) 74(3):283–96. 10.1007/s40265-014-0187-7 24554373 PMC4558097

[8] Ojangba T Boamah S Miao Y Guo X Fen Y Agboyibor C Comprehensive effects of lifestyle reform, adherence, and related factors on hypertension control: a review. J Clin Hypertens (Greenwich). (2023) 25(6):509–20. 10.1111/jch.14653 37161520 PMC10246465

[9] Tsai HY Chuang HJ Liao WH Wang YJ Li PH Wang WT Lifestyle modifications and non-pharmacological management in elderly hypertension. J Formosan Med Assoc. (2024) 124(Suppl 1):S32–S41. 10.1016/j.jfma.2024.10.022 39505584

[10] Cífková R. Hypertension in pregnancy: a diagnostic and therapeutic overview. High Blood Press Cardiovasc Prev. (2023) 30(4):289–303. 10.1007/s40292-023-00582-5 37308715 PMC10403432

[11] Odigboegwu O Pan LJ Chatterjee P. Use of antihypertensive drugs during preeclampsia. Front Cardiovasc Med. (2018) 5:50. 10.3389/fcvm.2018.00050 29896480 PMC5987086

[12] Yousofzai BS Walizada K Mehmood R Latoui RB Subhan M Espiegle E Maternal and neonatal outcomes in pregnant women with chronic hypertension: a retrospective study of 50 cases. Cureus. (2024) 16(9):e70316. 10.7759/cureus.70316 39469348 PMC11513204

[13] Bajpai D Popa C Verma P Dumanski S Shah S. Evaluation and management of hypertensive disorders of pregnancy. Kidney360. (2023) 4(10):1512–25. 10.34067/KID.0000000000000228 37526641 PMC10617800

[14] Ernawati U Wihastuti TA Utami YW. Effectiveness of diabetes self-management education (DSME) in type 2 diabetes mellitus (T2DM) patients: systematic literature review. J Public Health Res. (2021) 10(2):2240. 10.4081/jphr.2021.2240 33855427 PMC8129774

[15] Dailah HG. The influence of nurse-led interventions on diseases management in patients with diabetes Mellitus: a narrative review. Healthcare (Basel). (2024) 12(3):352. 10.3390/healthcare12030352 38338237 PMC10855413

[16] Baig AA Benitez A Quinn MT Burnet DL. Family interventions to improve diabetes outcomes for adults. Ann N Y Acad Sci. (2015) 1353(1):89–112. 10.1111/nyas.12844 26250784 PMC4624026

[17] McLean A Kirkham R Campbell S Whitbread C Barrett J Connors C Improving models of care for diabetes in pregnancy: experience of current practice in far north Queensland, Australia. Front Public Health. (2019) 7:192. 10.3389/fpubh.2019.00192 31380333 PMC6659099

[18] Yumei P Huiying K Liqin S Xiaoshan Z Meijing Z Yaping X The mediating effect of e-health literacy on social support and behavioral decision-making on glycemic management in pregnant women with gestational diabetes: a cross-sectional study. Front Public Health. (2024) 12:1416620. 10.3389/fpubh.2024.1416620 39086804 PMC11288816

[19] Villarreal-Granda P Recio-Platero A Martín-Bayo Y Durantez-Fernández C Cárdaba-García RM Pérez-Pérez L Models used by nurse case managers in different autonomous communities in Spain: a scoping review. Healthcare. (2024) 12(7):749. 10.3390/healthcare12070749 38610172 PMC11011987

